# Insights into the choice between intravenous infusion and subcutaneous injection: physician and patient characteristics driving treatment in SLE

**DOI:** 10.1007/s10067-020-05226-w

**Published:** 2020-07-04

**Authors:** Christopher F. Bell, Matthew Lau, Melody Lee, Christine Poulos

**Affiliations:** 1 GlaxoSmithKline, US Value, Evidence and Outcomes, Research Triangle Park, NC USA; 2 Present Address: Takeda Pharmaceuticals USA, Inc., Lexington, MA USA; 3 RTI Health Solutions, Research Triangle Park, NC USA

**Keywords:** Direct elicitation, Intravenous, Patient preference, Physician preference, Subcutaneous, Systemic lupus erythematosus

## Abstract

**Introduction/objectives:**

Multiple modes of administration are available for systemic lupus erythematosus (SLE) treatments. This study examined patient and physician characteristics associated with the choice of weekly subcutaneous (SC) injection or monthly intravenous (IV) infusion for an unspecified SLE treatment.

**Methods:**

This was a cross-sectional, US web-based survey using a direct elicitation, stated-preference methodology (HO-16-16706). Two hundred patients and 200 physicians were asked to choose between IV or SC administration in a hypothetical scenario. Pairwise and multivariate analyses estimated the odds ratio (OR) for the likelihood of choosing SC over IV for respondent characteristics.

**Results:**

Among patients, taking non-steroidal anti-inflammatory drugs increased the likelihood of choosing SC injection (OR 3.884), whilst having SLE-related skin problems, a fear of needles or self-injection, and never needing help around the house decreased the likelihood (OR 0.28, 0.13, 0.12, respectively; all *p* ≤ 0.05). Among physicians, > 95% recommended SC injection for patients who live or work far from an infusion center, prefer SC administration, and never or rarely miss medication doses. Physician characteristics including age and treatment practice also influenced choice.

**Conclusions:**

Patient and physician characteristics influence choice of SC versus IV therapy for SLE. These findings might inform shared decision-making, which could lead to improved patient outcomes.

**Table:** 

**Key Points** *• Data regarding patient and physician preference for different modes of administration of SLE therapy are sparse.* *• This cross-sectional*, *US web-based study showed that patient and physician characteristics influence choice of SC* versus *IV therapy for SLE.* *• A degree of disconnect exists between how factors influence patients*’ *choice and how those characteristics influence physicians*’ *choice of SLE treatment mode of administration.* *• The findings from this study might inform shared decision-making*, *which could improve alignment between treatment choice and patient preferences*, *treatment satisfaction*, *adherence*, *and improved patient outcomes.*

**Electronic supplementary material:**

The online version of this article (10.1007/s10067-020-05226-w) contains supplementary material, which is available to authorized users.

## Introduction

Systemic lupus erythematosus (SLE) is a chronic autoimmune disease characterized by diverse clinical manifestations and significant morbidity [1, 2]. Patients with SLE frequently experience periods of increased disease activity, termed flares, that contribute to the burden of the disease [3–5]. Furthermore, SLE is associated with a risk of organ-specific damage that increases steadily as the disease progresses [6].

Treatment strategies for SLE focus on managing symptoms, reducing disease activity, preventing organ damage, and improving health-related quality of life [7]. Current SLE treatment options are administered orally, or by intravenous (IV) infusion, or subcutaneous (SC) injection, with some treatments offering multiple administration options [8, 9]. SC administration is less invasive and takes less time than IV administration, which can improve patient convenience [10]. Additionally, SC therapies can be administered outside of the clinic, enabling patient independence, which is particularly important in long-term conditions such as SLE [10]. However, some patients prefer IV to SC administration, citing injection discomfort and the inconvenience of medication storage as reasons for their IV preference [11]. Belimumab, a human immunoglobulin G1λ monoclonal antibody against B lymphocyte stimulator (BLyS) [12], is an example of such a treatment, with IV and SC formulations available for the treatment of patients ages 5 years and older and 18 years and older, respectively, with active, autoantibody-positive SLE who are receiving standard therapy [13–16]. Since 2017 when belimumab became available as a SC formulation, many patients have been switched from IV to SC belimumab. One study showed that this transition was successful in all cases, with no impact on disease activity or patient satisfaction [17].

As recommended in recent European League Against Rheumatism (EULAR) guidelines, SLE care is multidisciplinary, based on a shared patient-physician decision, and should consider individual, medical, and societal costs [18]. Given the availability of multiple modes of administration for SLE treatments, choosing the most appropriate route for a particular patient should be a pivotal part of the shared decision-making process. Shared decision-making has been shown to be a useful tool in the management of chronic conditions [19–22], and may also play a key role in the management of SLE [22, 23]. Therefore, a thorough understanding of patient and physician factors driving preference for different modes of administration is of clinical relevance. This concept has been studied in a number of other therapy areas, including rheumatoid arthritis, severe asthma, and inflammatory bowel disease [24–28]. These studies revealed a number of factors that influence patient and physician decisions regarding the preference for mode of treatment administration, including age, hospital attendance, economic considerations, level of medical follow-up, injection frequency, convenience, and efficacy beliefs. However, few data are currently available regarding the preference for IV versus SC administration of SLE treatments from both the patient and physician perspective.

The aim of this study was to examine how patient and physician characteristics influenced preference for either IV infusion or SC injection of SLE treatment.

## Materials and methods

### Study design and assessments

This was a cross-sectional, web-based survey study (HO-16-16706) conducted with patient and physician respondents recruited from web panels in the United States of America (USA). The primary objective was to determine how respondent characteristics influenced patients’ and physicians’ choice of administration method between weekly SC injection and monthly IV infusion for an unspecified SLE treatment. The secondary objective was to calculate the likelihood that a patient with specific characteristics would choose a weekly SC versus a monthly IV administration of the same treatment.

**Fig. 1:**
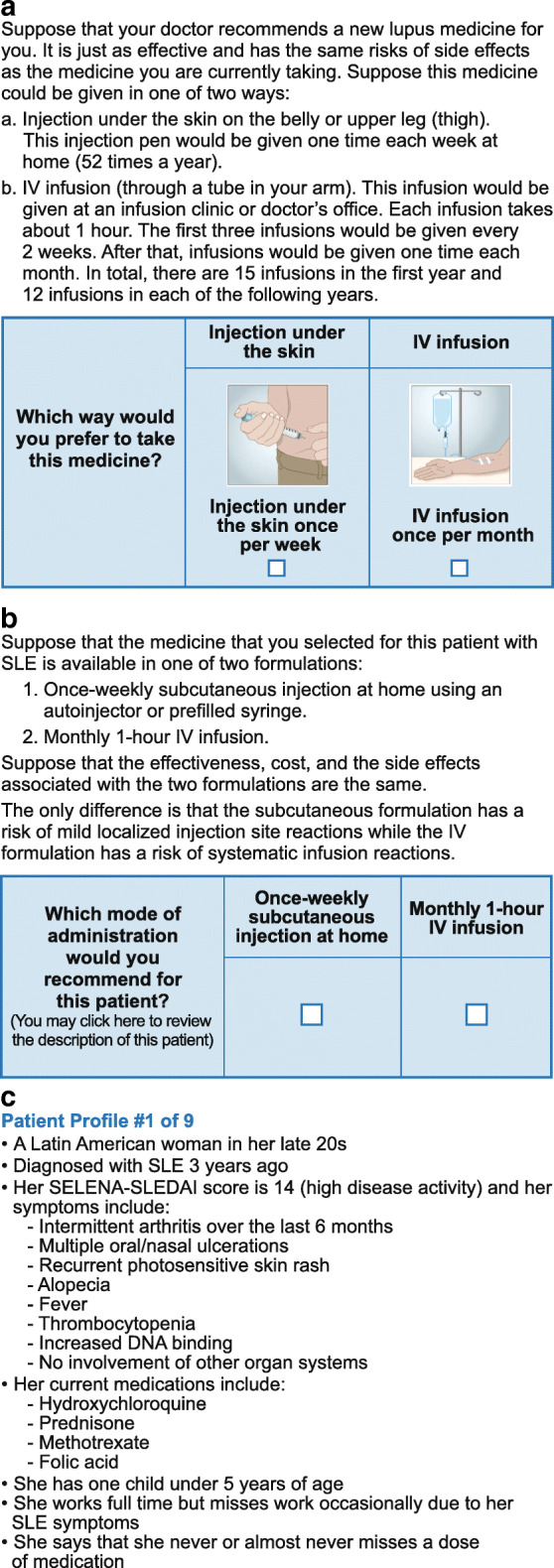
**a** Example direct elicitation question from the online patient survey. **b** Example direct elicitation question from the online physician survey. **c** Example patient profile from the online physician survey. DNA, deoxyribonucleic acid; IV, intravenous; SELENA-SLEDAI, Safety of Estrogens in Lupus Erythematosus National Assessment-Systemic Lupus Erythematosus Disease Activity Index; SLE, systemic lupus erythematosus

Respondents completed an online survey that used a stated-preference methodology (termed direct elicitation), whereby respondents were presented with a hypothetical scenario and asked to make a choice between IV or SC administration of a new, unspecified SLE medication. For patients, the survey described the method of IV and SC administration and asked one direct elicitation question: “Which way would you prefer to take your medication?” (Fig. 1A). The physician survey had two components. The first component examined how patient characteristics influence physician preference for IV or SC administration when each patient characteristic was explored independently. In this component, the survey asked physicians whether a series of patient characteristics (e.g., age, gender, employment status, type of health insurance) would influence their recommendation of weekly SC or monthly IV administration. If the physician indicated that a particular characteristic would influence their recommendation, the survey asked them whether they would be more likely to choose weekly SC or monthly IV administration for patients with that characteristic. The second component of the physician survey examined how physician preferences varied for each of a set of nine hypothetical patient profiles (Supplementary Table 1). Each profile was accompanied by a direct elicitation question: “Which mode of administration would you recommend for this patient?” It was assumed that the effectiveness, cost, and safety profile were the same, except that SC administration has a risk of mild injection site reactions and IV administration has a risk of systemic infusion reactions (Fig. 1B and C).

The study complied with all applicable laws, regulations, and guidance regarding patient protection, including patient privacy, and was reviewed by an institutional review board. Informed consent was obtained from all respondents.

### Survey development

Development of a draft survey was informed by the results of eight 45-min qualitative interviews with board-certified, practicing rheumatologists who treated patients with SLE. Half of the interviews were conducted in person and half by telephone. Of the eight physicians interviewed, three had in-house infusion capability and one was female. The concepts elicited in the physician interviews, together with a review of product labels and a targeted literature review of SLE treatment outcomes, were used to develop the patient and physician surveys.

The draft surveys were pretested using in-person semi-structured interviews with convenience samples of 15 patients with a self-reported diagnosis of SLE and 15 SLE-treating physicians. The objective of the interviews was to ensure that the items, response options, and recall periods were understandable and easily answered by respondents. Respondents were asked to think out loud as they completed the survey and were then asked a series of debriefing questions to determine whether they understood the definitions and instructions, accepted the hypothetical nature of the survey, and successfully completed the choice question in the survey. The survey was then finalized prior to online administration.

### Respondent populations

Patients and physicians were recruited by Survey Sampling International from a US web panel. For patients, the survey opportunity was posted on the panel website and interested panel members accessed the survey by clicking on the link. Physician panel members were directly invited to participate. Potential respondents were screened according to the following eligibility criteria: age ≥ 18 years with a self-confirmed diagnosis of SLE and resident in the USA. Physicians were board-certified or board-eligible rheumatologists residing in the USA who were currently treating patients with SLE each week.

### Data analysis

Data from all respondents who provided consent, answered ≥ 1 mode of administration direct elicitation choice question, and did not complete the survey too quickly (i.e., spent more than 6 min, based on an algorithm that determined the threshold survey length as a pre-specified fraction of the sample’s mean survey length in minutes) were included in the analysis.

The first stage of the analysis used pairwise comparisons to explore the relationship between patient characteristics and patient or physician preference for SC or IV administration. Patient characteristics were regressed on patient choice of administration mode in a series of logit regression models. Each regression included an intercept and a single patient characteristic. A similar analysis regressed patient characteristics on physician choice of mode.

The second stage of the analysis used three alternative multivariate analysis models to explore the relationship between respondent (patient or physician) characteristics and respondent preference for SC or IV administration mode. The responses to the direct elicitation question were used in separate logit regression models for physicians and patients. Independent variables included patient or physician respondent characteristics from each survey. For patients, the independent variables included demographic traits, socioeconomic status, and measures of disease severity and treatment. For physicians, the independent variables included demographic traits and training and practice characteristics. The physician models also included the hypothetical patient profiles as explanatory variables. Model specifications of the multivariate logistic regressions were informed by the pairwise Spearman correlation coefficients to identify pairs of variables likely to be correlated; only one of each pair of candidate independent variables with a statistically significant (*p* < 0.05) Spearman rank coefficient of ≥ 0.40 was included in the model.

Odds ratios (OR) were calculated for the pairwise comparisons and multivariate analyses of the likelihood of choosing SC injection over IV infusion for a variety of respondent characteristics (OR > 1 = increased likelihood of choosing SC administration; OR < 1 = reduced likelihood of choosing SC administration). The analyses identified several characteristics that had a statistically significant effect (*p* ≤ 0.05) on administration mode choice.

Finally, the average probability that a hypothetical patient in the sample would choose a weekly SC administration of an SLE treatment over a monthly IV administration was predicted using the results from each of the multivariate logistic models estimated with the patient data. These probabilities were calculated for hypothetical patients matching five of the nine hypothetical profiles from the physician survey. To perform these predictions, respondent characteristics measured in the patient survey were mapped to the selected patient characteristics in the hypothetical profiles.

## Results

### Respondent populations

Of 349 patients accessing the online survey, 219 were eligible and provided informed consent; of these, 200 completed the survey and were included in the final patient population. Demographics and clinical characteristics are shown in Table 1. A total of 6978 physicians were invited to participate, and 390 accessed the online survey. Of these, 264 were eligible and provided informed consent; 30 did not complete the survey, 7 completed it after the target sample size was reached, and a further 27 were excluded in an attempt to include an adequate number of physicians without infusion facilities. The remaining 200 participants were included in the final physician population; physician respondent characteristics are shown in Table 2.

**Table 1:** Patient respondent demographics and clinical characteristics

	All patients (*N* = 200)
Female, *n* (%)	172 (86.0)
Age, years, mean (SD)	50.4 (14.0)
Years since diagnosis, mean (SD)	13.2 (10.7)
SLE medications ever taken, *n* (%)	
NSAIDs	158 (79.0)
Antimalarial drugs	122 (61.0)
Steroid tablets or pills	128 (64.0)
Immunosuppressants	78 (39.0)
Biologics	23 (11.5)
Other	33 (16.5)
None	6 (3.0)
SLE medications currently taken, *n* (%)	
NSAIDs	102 (51.0)
Antimalarial drugs	88 (44.0)
Steroid tablets or pills	55 (27.5)
Immunosuppressants	40 (20.0)
Biologics	14 (7.0)
Other	25 (12.5)
None	19 (9.5)
Respondents who had ever experienced a SLE flare, *n* (%)	185 (92.5)
Respondents currently experiencing a SLE flare*, *n* (%)	64 (34.6)
Frequency of needing assistance to do things around the house†, *n* (%)	
Never	61 (30.5)
Rarely	26 (13.0)
Some of the time	73 (36.5)
Most of the time	28 (14.0)
All of the time	12 (6.0)

*NSAID* non-steroidal anti-inflammatory drug, *SD* standard deviation, *SLE* systemic lupus erythematosus

*Among patients who had ever experienced a SLE flare; †including cooking, cleaning, and washing clothes

**Table 2:** Physician respondent information

	All physicians (*N* = 200)
Female, *n* (%)	51 (25.5)
Age, years, mean (SD)	51.4 (10.9)
Average number of patients with SLE treated each week, *n* (%)	
≤ 5	16 (8.0)
6–10	59 (29.5)
11–20	65 (32.5)
21–30	28 (14.0)
31–40	17 (8.5)
41–50	5 (2.5)
> 50	10 (5.0)
Total years in practice since completion of medical training, *n* (%)	
< 10	43 (21.5)
10–15	50 (25.0)
16–20	24 (12.0)
21–25	35 (17.5)
> 25	48 (24.0)

*SD* standard deviation, *SLE* systemic lupus erythematosus

### Pairwise and multivariate analysis

#### Patient respondents

**Fig. 2:**
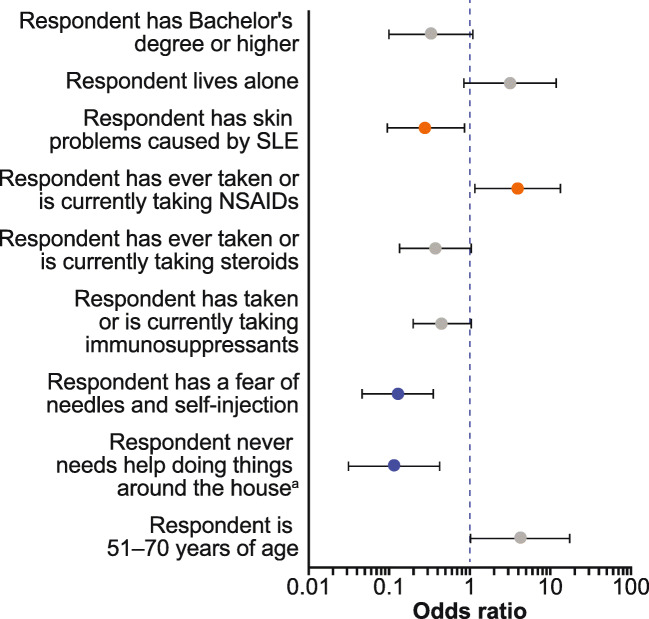
Multivariate analysis of patient responder surveys: variables associated with the likelihood of selecting SC injection mode of administration. ^a^Includes cooking, cleaning, and washing clothes. Grey circles denote values with *p* ≤ 0.1, orange circles denote values with *p* ≤ 0.05, blue circles denote values with *p* ≤ 0.01. Variables with an odds ratio > 1 indicate a characteristic that increased the likelihood of choosing SC injection administration, whereas an odds ratio < 1 indicate a characteristic that decreased the odds of choosing SC injection administration. Error bars represent 95% confidence intervals. NSAIDs, non-steroidal anti-inflammatory drugs; SC, subcutaneous; SLE, systemic lupus erythematosus

Multivariate analysis of patient responses indicated that factors associated with a higher likelihood of selecting SC administration were being 51–70 years of age rather than < 51 years or > 70 years of age (OR 4.18; *p* ≤ 0.10) and having ever taken or currently taking non-steroidal anti-inflammatory drugs (NSAIDs) rather than never having taken NSAIDs (OR 3.88; *p* ≤ 0.05) (Fig. 2). Conversely, characteristics most strongly associated with significantly lower likelihood of selecting SC administration over IV infusion were as follows: having SLE-related skin problems (OR 0.28; *p* ≤ 0.05), compared with not having SLE-related skin problems; having a fear of needles or self-injection (OR 0.13; *p* ≤ 0.01), compared with not being afraid, and never needing help doing household chores such as cooking and cleaning (OR 0.12; *p* ≤ 0.01), compared with needing assistance (Fig. 2). The results of the patient multivariate analysis were consistent with the results of the patient pairwise analysis (data not shown).

#### Physician respondents

The pairwise analysis of physician-stated preferences showed that five patient characteristics were considered by ≥ 75% of physicians to be deciding factors when recommending either SC or IV, and a further seven characteristics were considered to be deciding factors by > 50% of physicians (Supplementary Table 2). Of these physicians, a high proportion would recommend SC over IV administration for patients who live or work far from an infusion center (98.9%), prefer SC administration (97.8%), are employed (93.3%), have an active lifestyle (91.8%), almost never leave home (81.7%), never or rarely miss doses of medication (96.9%), prefer autonomy and independence in treatment matters (95.3%), or have experienced a severe infusion reaction (86.5%) (Supplementary Table 2). In contrast, physicians influenced by patient characteristics would recommend IV over SC administration for patients who regularly miss or skip medication doses (94.7%), prefer IV treatments (94.4%), are afraid of needles or self-injection (94.0%), or have Medicare insurance (89.5%) (Supplementary Table 2).

Physicians preferred SC administration for five out of nine hypothetical patient profiles (patients 1, 3, 5, 7, and 9), IV administration for three patient profiles (patients 4, 6, and 8), and were evenly split for SC and IV for patient profile 2 (Supplementary Table 3). SC administration was selected by ≥ 75% of physicians for four patient profiles; there was no patient profile for which ≥ 75% of physicians selected IV administration.

**Fig. 3:**
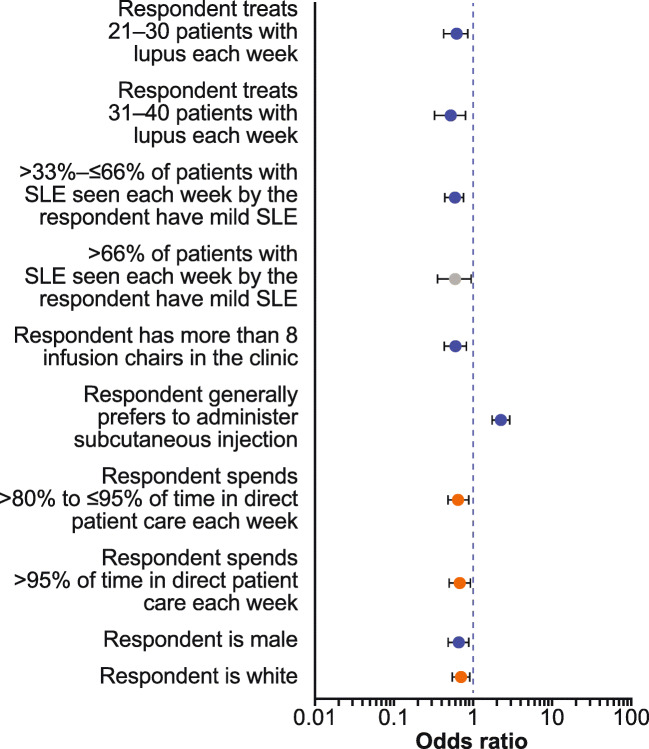
Multivariate analysis of physician responder surveys: variables associated with the odds of selecting SC injection mode of administration. Grey circles denote values with *p* ≤ 0.1, orange circles denote values with *p* ≤ 0.05, and blue circles denote values with *p* ≤ 0.01. Variables with an odds ratio > 1 indicate a characteristic that increased the likelihood of choosing SC injection administration, whereas an odds ratio < 1 indicate a characteristic that decreased the likelihood of choosing SC injection administration. Error bars represent 95% confidence intervals. SC, subcutaneous; SLE, systemic lupus erythematosus

In the multivariate analysis, physicians with a general preference for SC injections rather than IV infusion were significantly more likely to select SC injection over IV infusions as the form of administration (OR 2.32; *p* ≤ 0.01) (Fig. 3). In contrast, physician characteristics most significantly associated with a lower likelihood of selecting SC than IV administration were as follows: treating 21–30 (OR 0.62; *p* ≤ 0.01) or 31–40 (OR 0.53; *p* ≤ 0.01) patients, compared with treating < 11 patients or ≥ 41 patients with SLE per week; treating a population in which > 33–≤ 66% of patients have mild SLE (OR 0.59; *p* ≤ 0.01), compared with fewer or more patients with mild SLE; having > 8 infusion chairs in their office/suite (OR 0.62; *p* ≤ 0.01), compared with ≤ 8; and being male (OR 0.66; *p* ≤ 0.01) rather than female (Fig. 3).

### Predicted choice probabilities

Across the three multivariate models, patients similar to patient profile 6, a Latin American woman in her early 20s with moderate disease activity, no children, and a full-time job, were least likely to choose SC administration over IV administration. Patients similar to patient profile 4, an Asian woman in her mid-50s with high disease activity who cares for a family member and works part time, were most likely to choose SC over IV administration (Table 3).

**Table 3:** Average predicted probability of patients choosing weekly SC administration rather than monthly IV administration according to five hypothetical patient profiles^*†^

	Patient 1	Patient 2	Patient 4	Patient 6	Patient 7
	• A Latin American woman in her late 20s	• A Caucasian woman in her late 30s	• An Asian woman in her mid-50s	• A Latin American woman in her early 20s	• A Caucasian woman in her mid-30s
	• Diagnosed with SLE 3 years ago	• Diagnosed with SLE 5 years ago	• Diagnosed with SLE 8 years ago	• Diagnosed with SLE 3 years ago	• Diagnosed with SLE 5 years ago
	• Her SELENA-SLEDAI score is 14 (high disease activity) and her symptoms include:	• Her SELENA-SLEDAI score is 6 (moderate disease activity) and her symptoms include:	• Her SELENA-SLEDAI score is 12 (high disease activity) and her symptoms include:	• Her SELENA-SLEDAI score is 10 (moderate disease activity) and her symptoms include:	• Her SELENA-SLEDAI score is 6 (moderate disease activity) and her symptoms include:
	• Intermittent arthritis over the last 6 months	• Persistent polyarthritis	• Fatigue	• Arthritis diagnosed 10 months ago, now experiencing exacerbation	• Persistent polyarthritis involving the knee, and the 2nd and 3rd interphalangeal joints bilaterally
	• Multiple oral/nasal ulcerations	• Persistent malar rash	• 4 tender and swollen joints	• Multiple and recurrent oral ulcerations for the past 4 months	• Multiple recurrent oral ulcerations
	• Recurrent photosensitive skin rash	• Chronic fatigue	• Psychosis	• Low complement	• Fatigue
	• Alopecia	• No involvement of other organ systems	• No involvement of other organ systems	• Fever	• No involvement of other organ systems
	• Fever	• Her current medications include:	• Her current medications include:	• Leukopenia	• Her current medications include:
	• Thrombocytopenia	• Hydroxychloroquine	• Hydroxychloroquine	• No involvement of other organ systems	• Prednisone
	• Increased DNA binding	• Prednisone	• Prednisone	• Her current medications include:	• She has a child less than 5 years of age
	• No involvement of other organ systems	• She has teenaged children	• She has no children but cares for a family member	• Hydroxychloroquine	• She works full time
	• Her current medications include:	• She works full time	• She works part time	• Prednisone	• She says that she never or almost never misses a dose of medication
	• Hydroxychloroquine	• She says that she misses or skips some doses of her medications on a regular basis	• She says that she misses or skips some doses of her medications on a regular basis	• Azathioprine	
	• Prednisone			• She has no children	
	• Methotrexate			• She works full time but misses work occasionally due to her SLE symptoms	
	• Folic acid			• She says that she misses or skips some doses of her medications on a regular basis	
	• She has one child under 5 years of age				
	• She works full time but misses work occasionally due to her SLE symptoms				
	• She says that she never or almost never misses a dose of medication				
Model 1 probability (95% CI)	10.3 (− 11.6, 32.2)	35.8 (− 8.7, 80.3)	42.2 (0.4, 84.1)^†^	5.0 (− 6.6, 16.6)	28.7 (− 9.0, 66.5)
Model 2 probability (95% CI)	4.5 (− 6.4, 15.3)	27.2 (− 7.5, 62.0)	38.4 (− 1.9, 78.8)	2.2 (− 3.1, 7.5)	21.8 (− 8.3, 51.9)
Model 3 probability (95% CI)	10.2 (− 9.8, 30.2)	16.0 (− 17.6, 49.7)	31.3 (− 21.2, 83.7)	2.2 (− 3.5, 7.9)	26.9 (− 5.4, 59.3)

*CI* confidence interval, *IV* intravenous, *SC* subcutaneous, *SELENA-SLEDAI* Safety of Estrogens in Lupus Erythematosus National Assessment-Systemic Lupus Erythematosus Disease Activity Index, *SLE* systemic lupus erythematosus

*Predicted choices are based on patients that are as similar as possible to the hypothetical patient profiles; however, patients did not match the hypothetical profiles exactly; ^†^statistically significant at the 95% level of confidence

## Discussion

This exploratory preference study is among the first to include patients with SLE and physicians who treat patients with SLE. Findings suggest that a range of patient and physician characteristics might play a role in choosing one mode of administration over another. Patient characteristics influencing their choice of once-weekly SC self-injection at home over monthly IV infusion at a healthcare center were receipt of current or previous NSAID treatment and being aged 51–70 years. In line with this, the hypothetical patient profile associated with the highest probability of choosing SC over IV administration was for a woman in her mid-50s. Notably, patients with a profile similar to the hypothetical patient with high disease activity (patient 4) were most likely to choose SC over IV administration, which may indicate a preference for SC administration in patients with high disease activity. Patients with more severe disease, including those with SLE-related skin problems, and those with a fear of needles, were more inclined to choose IV administration; again, these findings were supported by the predicted choice probability exercise. When considering patient characteristics that influenced physician choice of administration mode, sociodemographic factors such as patient age, gender, and race were generally not influencers, nor were clinical characteristics such as symptoms, time since diagnosis, disease activity, and treatment. This might suggest that the factors influencing patient choice differ from the factors that influence physician choice. Physicians considered distance from an infusion center, patient preference for SC or IV, patient lifestyle, adherence to medication, and attitude towards treatment decisions as important factors. Physicians were more likely to recommend SC administration for patients with an active lifestyle and those with good adherence to medication. When considering choice of administration mode for hypothetical patient profiles, of the five patients for whom physicians selected SC administration, all had good medication adherence and all but one worked or had an active lifestyle. However, the age of the patients, time since diagnosis, and disease activity varied. The key characteristic that affected the physician’s decision to recommend SC over IV administration was a general preference to administer treatment by the SC route rather than the IV route. Characteristics influencing physicians to recommend IV over SC included treating 21–40 patients with SLE each week, treating a patient population in which more than one third have mild SLE, having > 8 infusion chairs, and being male.

Non-adherence to treatment is a substantial problem in SLE. A systematic literature review reported that 43–75% of patients with SLE are non-adherent to treatment, with most studies in the review reporting more than 50% non-adherence [29]. Lower education level, depression, and polypharmacy were consistently reported as factors associated with non-adherence [29]. Several studies have demonstrated that non-adherence with therapy is associated with poor outcomes in SLE [30–32]. Shared decision-making is associated with improved adherence [33], and a better understanding of factors that influence choice of administration mode by patients and physicians may ultimately help to improve adherence rates, and therefore, outcomes, in SLE. Indeed, patients with SLE who participate more actively in physician–patient consultations have been shown to accrue less organ damage over a ~ 5-year period [34]. In addition, therapeutic levels of SLE treatments in the blood, which can provide a measure of adherence, have been associated with decreased disease activity compared with undetectable or subtherapeutic levels [35]. Furthermore, the study by Durcan et al. demonstrated that with routine blood testing and patient counselling, adherence can be significantly improved. Data from these studies highlight the real clinical importance of joint decision-making involving both the patient and physician to ensure that the patient receives the most appropriate treatment, ultimately leading to improved adherence and both short-term and long-term clinical benefits.

Previous studies from other therapy areas, including rheumatoid arthritis, severe asthma, and inflammatory bowel disease, have revealed several patient and physician decisions that influence the preference for IV versus SC administration methods [24–28]; however, minimal data are available for SLE therapies. The findings of our study build upon previous studies investigating patient preference for IV versus SC administration of SLE treatment. A survey of patients in Italy evaluating preferences for administration of biological therapy showed that 41% preferred SC administration, 37% preferred IV infusion, and 22% were uncertain [36]. Patients who preferred SC administration were primarily motivated by convenience, whilst those preferring IV administration were motivated by the safety of having healthcare professionals present during treatment [36]. In an analysis of the Lupus Plus Project, which reported real-world data from patients receiving belimumab in the USA, 51% of patients stated that they would prefer to self-administer belimumab at home, 20% preferred IV administration at a clinic, and 29% had no preference [37]. A study among patients who switched from belimumab IV to self-administering belimumab using the autoinjector found that the majority (76%) preferred treatment with the autoinjector, considering it to be more convenient than belimumab IV due to shorter administration time, less travel time, less interference with work, portability, and reduced/no pain [11].

This study has a number of strengths; namely, the surveys were carefully designed and were pretested using in-depth interviews with patients with self-reported diagnosis of SLE and with physicians who treat patients with SLE. However, there are several potential limitations of this type of assessment. One inherent limitation is that the respondents evaluated hypothetical treatments, and their choices did not have the same significance as choices involving actual treatment decisions. Actual treatment choices may depend on several contextual factors that were beyond the scope of this study. Another limitation is that the study used a convenience sample of patients recruited through a web-based panel, which might not be representative of the overall populations of patients with SLE and physicians treating SLE in the USA. Moreover, the influence of potential language barriers for patients or physicians conducting the surveys was not determined. In addition, information regarding SLE diagnoses and respondent characteristics was self-reported, and it is possible that some patients who completed the study did not have SLE. Furthermore, the primary objective of the study was descriptive; as such, the results of the study cannot be formally validated.

In conclusion, the insights from this study into the characteristics influencing patient and physician preferences for different modes of administration of SLE treatment may inform shared decision-making, which may lead to better alignment between treatment choice and patient preferences, treatment satisfaction, adherence, and improved patient outcomes.

## Electronic supplementary material

ESM 1 (DOCX 31.9 kb).

## Data Availability

GSK is committed to publicly disclosing the results of GSK-sponsored clinical research that evaluates GSK medicines, and as such was involved in the decision to submit. Study documents can be requested for further research from www.clinicalstudydatarequest.com. Researchers can inquire about the availability of data from GSK clinical studies that are not listed on the site before they submit a research proposal.

## References

[1] Kuhn A, Bonsmann G, Anders H-J, Herzer P, Tenbrock K, Schneider M (2015). The diagnosis and treatment of systemic lupus erythematosus. Dtsch Arztebl Int.

[2] Manson JJ, Rahman A (2006). Systemic lupus erythematosus. Orphanet J Rare Dis.

[3] Fernandez D, Kirou KA (2016). What causes lupus flares?. Curr Rheumatol Rep.

[4] Garris C, Jhingran P, Bass D, Engel-Nitz NM, Riedel A, Dennis G (2013). Healthcare utilization and cost of systemic lupus erythematosus in a US managed care health plan. J Med Econ.

[5] Kan H, Guerin A, Kaminsky MS, Yu AP, Wu EQ, Denio A, Priti J, Narayanan S, Molta C (2013). A longitudinal analysis of costs associated with change in disease activity in systemic lupus erythematosus. J Med Econ.

[6] Chambers SA, Allen E, Rahman A, Isenberg D (2009). Damage and mortality in a group of British patients with systemic lupus erythematosus followed up for over 10 years. Rheumatology (Oxford).

[7] Touma Z, Gladman DD (2017). Current and future therapies for SLE: obstacles and recommendations for the development of novel treatments. Lupus Sci Med.

[8] Amissah-Arthur MB, Gordon C (2010). Contemporary treatment of systemic lupus erythematosus: an update for clinicians. Ther Adv Chronic Dis.

[9] European Medicines Agency (EMA) (2016) Benlysta summary of product characteristics. https://www.ema.europa.eu/documents/product-information/benlysta-epar-product-information_en.pdf. Accessed 14 February 2019

[10] Yapa SW, Roth D, Gordon D, Struemper H (2016). Comparison of intravenous and subcutaneous exposure supporting dose selection of subcutaneous belimumab systemic lupus erythematosus phase 3 program. Lupus.

[11] Dashiell-Aje E, Harding G, Pascoe K, DeVries J, Berry P, Ramachandran S (2018). Patient evaluation of satisfaction and outcomes with an autoinjector for self-administration of subcutaneous belimumab in patients with systemic lupus erythematosus. Patient.

[12] Baker KP, Edwards BM, Main SH, Choi GH, Wager RE, Halpern WG, Lappin PB, Riccobene T, Abramian D, Sekut L (2003). Generation and characterization of LymphoStat-B, a human monoclonal antibody that antagonizes the bioactivities of B lymphocyte stimulator. Arthritis Rheum.

[13] Food and Drug Administration (2019) BENLYSTA (belimumab) prescribing information. https://www.gsksource.com/pharma/content/dam/GlaxoSmithKline/US/en/Prescribing_Information/Benlysta/pdf/BENLYSTA-PI-MG-IFU-COMBINED.PDF. Accessed 11 September 2019

[14] Furie R, Petri M, Zamani O, Cervera R, Wallace DJ, Tegzová D, Sanchez-Guerrero J, Schwarting A, Merrill JT, Chatham WW (2011). A phase III, randomized, placebo-controlled study of belimumab, a monoclonal antibody that inhibits B lymphocyte stimulator, in patients with systemic lupus erythematosus. Arthritis Rheum.

[15] Navarra SV, Guzman RM, Gallacher AE, Hall S, Levy RA, Jimenez RE, Li EK, Thomas M, Kim HY, Leon MG, Tanasescu C, Nasonov E, Lan JL, Pineda L, Zhong ZJ, Freimuth W, Petri MA (2011). Efficacy and safety of belimumab in patients with active systemic lupus erythematosus: a randomised, placebo-controlled, phase 3 trial. Lancet.

[16] Stohl W, Schwarting A, Okada M, Scheinberg M, Doria A, Hammer AE, Kleoudis C, Groark J, Bass D, Fox NL (2017). Efficacy and safety of subcutaneous belimumab in systemic lupus erythematosus: a fifty-two–week randomized, double-blind, placebo-controlled study. Arthritis Rheum.

[17] Mucke J, Brinks R, Fischer-Betz R, Richter JG, Sander O, Schneider M, Chehab G (2019). Patient satisfaction and disease control in patients with systemic lupus erythematosus is not affected by switching from intravenous belimumab to subcutaneous injections. Patient Prefer Adherence.

[18] Fanouriakis A, Kostopoulou M, Alunno A, Aringer M, Bajema I, Boletis JN, Cervera R, Doria A, Gordon C, Govoni M, Houssiau F, Jayne D, Kouloumas M, Kuhn A, Larsen JL, Lerstrom K, Moroni G, Mosca M, Schneider M, Smolen JS, Svenungsson E, Tesar V, Tincani A, Troldborg A, van Vollenhoven R, Wenzel J, Bertsias G, Boumpas DT (2019). 2019 update of the EULAR recommendations for the management of systemic lupus erythematosus. Ann Rheum Dis.

[19] Desroches S (2010) Shared decision making and chronic diseases. Allergy Asthma Clin Immunol 6(Suppl 4):A8

[20] Gionfriddo MR, Leppin AL, Brito JP, Leblanc A, Boehmer KR, Morris MA, Erwin PJ, Prokop LJ, Zeballos-Palacios CL, Malaga G, Miranda JJ, McLeod HM, Rodriguez-Gutierrez R, Huang R, Morey-Vargas OL, Murad MH, Montori VM (2014). A systematic review of shared decision making interventions in chronic conditions: a review protocol. Syst Rev.

[21] Olomu A, Hart-Davidson W, Luo Z, Kelly-Blake K, Holmes-Rovner M (2016). Implementing shared decision making in federally qualified health centers, a quasi-experimental design study: the Office-Guidelines Applied to Practice (Office-GAP) program. BMC Health Serv Res.

[22] Qu H, Shewchuk RM, Alarcon G, Fraenkel L, Leong A, Dall'Era M, Yazdany J, Singh JA (2016). Mapping perceptions of lupus medication decision-making facilitators: the importance of patient context. Arthritis Care Res.

[23] Yedimenko J, Hackenberger P, Sullivan E, Morris K, Meara A (2018) Effectiveness of shared decision making in systemic lupus erythematosus patients at OSU. Arthritis Rheum:70

[24] Allen PB, Lindsay H, Tham TC (2010). How do patients with inflammatory bowel disease want their biological therapy administered?. BMC Gastroenterol.

[25] Bolge SC, Goren A, Brown D, Ginsberg S, Allen I (2016). Openness to and preference for attributes of biologic therapy prior to initiation among patients with rheumatoid arthritis: patient and rheumatologist perspectives and implications for decision making. Patient Prefer Adherence.

[26] Chilton F, Collett RA (2008). Treatment choices, preferences and decision-making by patients with rheumatoid arthritis. Musculoskeletal Care.

[27] Desplats M, Pascart T, Jelin G, Norberciak L, Philippe P, Houvenagel E, Goeb V, Flipo RM (2017). Are abatacept and tocilizumab intravenous users willing to switch for the subcutaneous route of administration? A questionnaire-based study. Clin Rheumatol.

[28] Santus P, Ferrando M, Baiardini I, Radovanovic D, Fattori A, Braido F (2019). Patients beliefs on intravenous and subcutaneous routes of administration of biologics for severe asthma treatment: a cross-sectional observational survey study. World Allergy Organ J.

[29] Mehat P, Atiquzzaman M, Esdaile JM, AviNa-Zubieta A, De Vera MA (2017) Medication nonadherence in systemic lupus erythematosus: a systematic review. Arthritis Care Res (Hoboken) 69:1706–1713. 10.1002/acr.23191 10.1002/acr.23191 28086003

[30] Feldman CH, Yazdany J, Guan H, Solomon DH, Costenbader KH (2015) Medication nonadherence is associated with increased subsequent acute care utilization among Medicaid beneficiaries with systemic lupus erythematosus. Arthritis Care Res (Hoboken) 67:1712–1721. 10.1002/acr.22636 10.1002/acr.22636 PMC4684806 26097166

[31] Julian LJ, Yelin E, Yazdany J, Panopalis P, Trupin L, Criswell LA, Katz P (2009). Depression, medication adherence, and service utilization in systemic lupus erythematosus. Arthritis Rheum.

[32] Rojas-Serrano J, Cardiel MH (2000). Lupus patients in an emergency unit. Causes of consultation, hospitalization and outcome. A cohort study. Lupus.

[33] Schoenthaler A, Rosenthal DM, Butler M, Jacobowitz L (2018). Medication adherence improvement similar for shared decision-making preference or longer patient-provider relationship. J Am Board Fam Med.

[34] Ward MM, Sundaramurthy S, Lotstein D, Bush TM, Neuwelt CM, Street RL (2003). Participatory patient-physician communication and morbidity in patients with systemic lupus erythematosus. Arthritis Rheum.

[35] Durcan L, Clarke WA, Magder LS, Petri M (2015). Hydroxychloroquine blood levels in systemic lupus erythematosus: clarifying dosing controversies and improving adherence. J Rheumatol.

[36] Falanga M, Canzona A, Mazzoni D (2019). Preference for subcutaneous injection or intravenous infusion of biological therapy among Italian patients with SLE. J Patient Exp.

[37] Pascoe K, Lobosco S, Bell D, Hoskin B, Chang DJ, Pobiner B, Ramachandran S (2017). Patient- and physician-reported satisfaction with systemic lupus erythematosus treatment in US clinical practice. Clin Ther.

