# Supplementary material for: Insights into the choice between intravenous infusion and subcutaneous injection: physician and patient characteristics driving treatment in SLE
Source: Clin Rheumatol. 2020 Jul 4;40(2):581–90. doi: 10.1007/s10067-020-05226-w (PMC7817604; doi:10.1007/s10067-020-05226-w)
Supplement: Supplementary file 1 — (DOCX 31.9 kb). [file 10067_2020_5226_MOESM1_ESM.docx]

**Supplementary Table 1** Hypothetical patient profiles included in the physician survey

| Patient | Profile description |
| --- | --- |
| Patient 1 | - A Latin American woman in her late 20s - Diagnosed with SLE 3 years ago - Her SELENA-SLEDAI score is 14 (high disease activity) and her symptoms include: - Intermittent arthritis over the last 6 months - Multiple oral/nasal ulcerations - Recurrent photosensitive skin rash - Alopecia - Fever - Thrombocytopenia - Increased DNA binding - No involvement of other organ systems - Her current medications include: - Hydroxychloroquine - Prednisone - Methotrexate - Folic acid - She has one child under 5 years of age - She works full time but misses work occasionally due to her SLE symptoms - She says that she never or almost never misses a dose of medication |
| Patient 2 | - A Caucasian woman in her late 30s - Diagnosed with SLE 5 years ago - Her SELENA-SLEDAI score is 6 (moderate disease activity) and her symptoms include: - Persistent polyarthritis - Persistent malar rash - Chronic fatigue - No involvement of other organ systems - Her current medications include: - Hydroxychloroquine - Prednisone - She has teenaged children - She works full time - She says that she misses or skips some doses of her medications on a regular basis |
| Patient 3 | - A Caucasian woman in her early 40s - Diagnosed with SLE 10 years ago - Her SELENA-SLEDAI score is 14 (high disease activity) and her symptoms include: - Polyarthritis involving knees, wrists, and hands - Inflammatory skin rash - Increasing fatigue - Persistent myalgia - Increased DNA binding - Low complement - Proteinuria - No involvement of other organ systems - Her current medications include: - Hydroxychloroquine - Prednisone - Mycophenolate mofetil - NSAIDs as needed - She is not active - She is disabled and unable to work - She says that she never or almost never misses a dose of medication |
| Patient 4 | - An Asian woman in her mid-50s - Diagnosed with SLE 8 years ago - Her SELENA-SLEDAI score is 12 (high disease activity) and her symptoms include: - Fatigue - 4 tender and swollen joints - Psychosis - No involvement of other organ systems - Her current medications include: - Hydroxychloroquine - Prednisone - She has no children but cares for a family member - She works part time - She says that she misses or skips some doses of her medications on a regular basis |
| Patient 5 | - A Caucasian male in his mid-20s - Diagnosed with SLE 5 years ago - His SELENA-SLEDAI score is 14 (high disease activity) and his symptoms include: - Seizure - Mucosal ulcers - Myalgia - Pyuria - No involvement of other organ systems - His current medications include: - Hydroxychloroquine - Prednisone - Mycophenolate mofetil - He has no children - He is a student - He says that he never or almost never misses a dose of medication |
| Patient 6 | - A Latin American woman in her early 20s - Diagnosed with SLE 3 years ago - Her SELENA-SLEDAI score is 10 (moderate disease activity) and her symptoms include: - Arthritis diagnosed 10 months ago, now experiencing exacerbation - Multiple and recurrent oral ulcerations for the past 4 months - Low complement - Fever - Leukopenia - No involvement of other organ systems - Her current medications include: - Hydroxychloroquine - Prednisone - Azathioprine - She has no children - She works full time but misses work occasionally due to her SLE symptoms - She says that she misses or skips some doses of her medications on a regular basis |
| Patient 7 | - A Caucasian woman in her mid-30s - Diagnosed with SLE 5 years ago - Her SELENA-SLEDAI score is 6 (moderate disease activity) and her symptoms include: - Persistent polyarthritis involving the knee, and the 2nd and 3rd interphalangeal joints bilaterally - Multiple recurrent oral ulcerations - Fatigue - No involvement of other organ systems - Her current medications include: - Prednisone - She has a child under 5 years of age - She works full time - She says that she never or almost never misses a dose of medication |
| Patient 8 | - An African-American woman in her early 40s - Diagnosed with SLE 10 years ago - Her SELENA-SLEDAI score is 14 (high disease activity) and her symptoms include: - Polyarthritis involving the knees, wrists, and hands - Inflammatory skin rash - Increasing fatigue - Persistent myalgia - Hematuria - Urinary casts - No involvement of other organ systems - Her current medications include: - Hydroxychloroquine - Mycophenolate mofetil - NSAIDs - She has no children, and is not active outside of work - She works full time but misses work occasionally due to her SLE symptoms - She says that she misses or skips some doses of her medications on a regular basis |
| Patient 9 | - An African-American woman in her mid-50s - Diagnosed with SLE 8 years ago - Her SELENA-SLEDAI score is 10 (moderate disease activity) and her symptoms include: - Malar rash - 4 tender and swollen joints - Periungual erythema - Myositis - No involvement of other organ systems - Her current medications include: - Hydroxychloroquine - Prednisone - She is active in her community - She is unemployed - She says that she never or almost never misses a dose of medication |

*DNA* deoxyribonucleic acid, *NSAID* non-steroidal anti-inflammatory drug, *SELENA-SLEDAI* Safety of Estrogens in Lupus Erythematosus National Assessment-Systemic Lupus Erythematosus Disease Activity Index, *SLE* systemic lupus erythematosus

**Supplementary Table 2** Physician responses regarding the impact of patient characteristics on the choice between SC and IV administration mode

| Patient demographic and socioeconomic characteristics (n = 200 physician responses unless otherwise stated) | Would this characteristic be a deciding factor in which formulation (SC or IV) the physician would recommend? | | If yes, which formulation would the physician recommend for a patient with SLE with this characteristic? | |
| --- | --- | --- | --- | --- |
|  | Yes, n (%) | No, n (%) | Once-weekly SC injection at Home, n (%) | Monthly 1-hour IV infusion, n (%) |
| Gender: Man (n = 199) | 19 (9.6) | 180 (90.5) | 6 (31.6) | 13 (68.4) |
| Gender: Woman | 12 (6.0) | 188 (94.0) | 6 (50.0) | 6 (50.0) |
| Race/ethnicity: Latin American | 20 (10.0) | 180 (90.0) | 2 (10.0) | 12 (90.0) |
| Race/ethnicity: White/Caucasian (n = 199) | 10 (5.0) | 189 (95.0) | 6 (60.0) | 4 (40.0) |
| Race/ethnicity: Asian (n = 198) | 12 (6.1) | 186 (93.9) | 7 (58.3) | 5 (41.7) |
| Race/ethnicity: African American | 28 (14.0) | 172 (86.0) | 3 (10.7) | 25 (89.3) |
| Less than 18 years of age (n = 199) | 80 (40.2) | 119 (59.8) | 26 (32.5) | 54 (67.5) |
| Age 18–29 years | 60 (30.0) | 140 (70.0) | 30 (50.0) | 30 (50.0) |
| Age 30–39 years (n = 199) | 28 (14.1) | 171 (85.9) | 24 (85.7) | 4 (14.3) |
| Age 40–49 years (n = 198) | 22 (11.1) | 176 (88.9) | 19 (86.4) | 3 (13.6) |
| Age 50–59 years (n = 199) | 22 (11.1) | 177 (88.9) | 13 (59.1) | 9 (40.1) |
| Age 60–69 years (n = 199) | 66 (33.2) | 133 (66.8) | 8 (12.1) | 58 (87.9) |
| Age 70+ years (n = 198) | 96 (48.5) | 102 (51.5) | 9 (9.4) | 87 (90.6) |
| Type of health insurance: Medicare | 125 (62.8)  (n = 199) | 74 (37.2)  (n = 199) | 13 (10.5)  (n = 198) | 111 (89.5)  (n = 198) |
| Employment status: employed | 105 (52.5) | 95 (47.5) | 98 (93.3) | 7 (6.7) |
| Employment status: disabled/unable to work | 61 (30.5) | 139 (69.5) | 16 (26.2) | 45 (73.8) |
| Employment status: student | 85 (42.5) | 115 (57.5) | 74 (87.1) | 11 (12.9) |
| Patient has an active lifestyle (has young children, active in community, cares for family member or friend, physically active, travels frequently, commutes a lot) (n = 199) | 134 (67.3) | 65 (32.7) | 123 (91.8) | 11 (8.2) |
| Patient has an inactive lifestyle (gets out only as needed) | 71 (35.5) | 129 (64.5) | 36 (51.4)  (n = 199) | 34 (48.6)  (n = 199) |
| Patient almost never leaves his or her home (n = 199) | 121 (60.8) | 78 (39.2) | 98 (81.7)  (n = 198) | 22 (18.3)  (n = 198) |
| Patient has support at home (for example, to help with treatment, transportation, errands, and self-care) (n = 199) | 85 (42.7) | 114 (57.3) | 60 (71.4) | 24 (28.6) |
| Patient lives and works far away from an infusion center (n = 199) | 187 (94.0) | 12 (6.0) | 185 (98.9) | 2 (1.1) |
| Patient lives or works nearby an infusion center (n = 199) | 63 (31.7) | 136 (68.3) | 6 (9.5) | 57 (90.5) |
| Patient never or rarely misses or skips doses (n = 199) | 129 (64.8) | 70 (35.2) | 124 (96.9)  (n = 198) | 4 (3.1)  (n = 198) |
| Patient misses or skips doses on a regular basis | 169 (84.5) | 31 (15.5) | 9 (5.3) | 160 (94.7) |
| Patient prefers SC (n = 199) | 180 (90.5) | 19 (9.5) | 176 (97.8) | 4 (2.2) |
| Patient prefers IV (n = 199) | 179 (89.9) | 20 (10.1) | 10 (5.6) | 169 (94.4) |
| Patient is afraid of needles and/or self-injection (n = 199) | 169 (84.9) | 30 (15.1) | 10 (6.0)  (n = 198) | 158 (94.0)  (n = 198) |
| Patient defers to health care providers for all matters related to treatment (including treatment choice and monitoring) (n = 199) | 60 (30.2) | 139 (69.9) | 22 (37.3)  (n = 198) | 37 (62.7)  (n = 198) |
| Patient prefers autonomy and independence in all matters related to treatment | 108 (54.0) | 92 (46.0) | 102 (95.3)  (n = 199) | 5 (4.7)  (n = 199) |
| Years since diagnosis: 1 year or less (n = 199) | 26 (13.1) | 173 (86.9) | 14 (53.8) | 12 (46.2) |
| Years since diagnosis: more than one year but less than 10 (n = 199) | 16 (8.0) | 183 (92.0) | 11 (68.8) | 5 (31.3) |
| Years since diagnosis: more than 10 years | 17 (8.5) | 183 (91.5) | 10 (58.8) | 7 (41.2) |
| Symptoms/manifestations: joint and muscle involvement (for example, arthritis, swollen and tender joints, myalgia) (n = 199) | 26 (13.1) | 173 (86.9) | 13 (50.0) | 13 (50.0) |
| Symptoms/manifestations: kidney involvement (n = 199) | 61 (30.7) | 138 (69.3) | 7 (11.5) | 54 (88.5) |
| Symptoms/manifestations: central nervous system involvement | 82 (41.0) | 118 (59.0) | 4 (4.9) | 78 (95.1) |
| Symptoms/manifestations: cardiovascular involvement (n = 199) | 49 (24.6) | 150 (75.4) | 4 (8.5)  (n = 197) | 43 (91.5)  (n = 197) |
| Symptoms/manifestations: pulmonary involvement (n = 199) | 52 (26.1) | 147 (73.9) | 6 (11.5) | 46 (88.5) |
| Symptoms/manifestations: blood and blood vessel involvement (for example, Raynaud’s phenomenon [fingers and toes turn blue when cold or stressed, turn red when blood returns]) | 36 (18.0) | 164 (82.0) | 10 (28.6)  (n = 199) | 25 (71.4)  (n = 199) |
| Symptoms/manifestations: constitutional symptoms (for example, fever, fatigue) (n = 199) | 21 (10.6) | 178 (89.4) | 9 (45.0)  (n = 198) | 11 (55.0)  (n = 198) |
| Symptoms/manifestations: skin involvement (for example, mouth ulcers, skin rashes [malar, photosensitive, discoid], alopecia, periungual erythema) | 21 (10.5) | 179 (89.5) | 13 (65.0)  (n = 199) | 7 (35.0)  (n = 199) |
| SELENA-SLEDAI score: 5 and under (mild disease activity) (n = 199) | 31 (15.6) | 168 (84.4) | 28 (90.3) | 3 (9.7) |
| SELENA-SLEDAI score: 6-10 (moderate disease activity) (n = 199) | 31 (15.6) | 168 (84.4) | 16 (51.6) | 15 (48.4) |
| SELENA-SLEDAI score: 11-19 (high disease activity) (n = 199) | 67 (33.7) | 132 (66.3) | 5 (7.5) | 62 (92.5) |
| SELENA-SLEDAI score: greater than 19 (very high disease activity) | 82 (41.0) | 118 (59.0) | 6 (7.3) | 76 (92.7) |
| Patient is experiencing a flare | 39 (19.5) | 161 (80.5) | 10 (25.6) | 29 (74.4) |
| Concomitant treatment: NSAIDs (n = 199) | 5 (2.5) | 194 (97.5) | 4 (80.0) | 1 (20.0) |
| Concomitant treatment: oral corticosteroids (n = 199) | 9 (4.5) | 190 (95.5) | 2 (22.2) | 7 (77.8) |
| Concomitant treatment: hydroxychloroquine (n = 199) | 5 (2.5) | 194 (97.5) | 3 (60.0) | 2 (40.0) |
| Concomitant treatment: immunosuppressants (for example, methotrexate, azathioprine, mycophenolate) | 26 (13.0) | 174 (87.0) | 5 (19.2) | 21 (80.8) |
| Previous treatment with hydroxychloroquine and prednisone, but still experiencing active disease | 23 (11.5) | 177 (88.5) | 10 (43.5) | 13 (56.5) |
| Previous treatment with hydroxychloroquine, prednisone, and an immunosuppressant, but still experiencing active disease (n = 199) | 33 (16.6) | 166 (83.4) | 4 (12.1) | 29 (87.9) |
| Patient is obese (n = 199) | 51 (25.6) | 148 (74.4) | 12 (23.5) | 39 (76.5) |
| Patient has previously experienced a severe infusion reaction (n = 199) | 133 (66.8) | 66 (33.2) | 115 (86.5) | 18 (13.5) |

*IV* intravenous, *NSAID* non-steroidal anti-inflammatory drug, *SC* subcutaneous, *SELENA-SLEDAI* Safety of Estrogens in Lupus Erythematosus National Assessment-Systemic Lupus Erythematosus Disease Activity Index, *SLE* systemic lupus erythematosus

**Supplementary Table 3** Physician preferences for mode of administration according to hypothetical patient profile

| **Patient profile** | **Physician preference, n (%)** | | **Degree of certainty, mean (SD)*** |
| --- | --- | --- | --- |
|  | **Once-weekly SC Injection at Home** | **Monthly 1-Hour IV Infusion** |  |
| Patient 1 | 163 (81.5) | 37 (18.5) | 5.9 (0.96) |
| Patient 2 | 100 (50.0) | 100 (50.0) | 5.7 (1.05) |
| Patient 3 | 140 (70.0) | 60 (30.0) | 5.7 (1.05) |
| Patient 4 | 54 (27.0) | 146 (73.0) | 5.8 (0.99) |
| Patient 5 | 154 (77.0) | 46 (23.0) | 5.8 (0.93) |
| Patient 6 | 74 (37.0) | 126 (63.0) | 5.7 (0.97) |
| Patient 7 | 181 (90.5) | 19 (9.5) | 6.0 (1.01) |
| Patient 8 | 65 (32.5) | 135 (67.5) | 5.8 (1.04) |
| Patient 9 | 159 (79.5) | 41 (20.5) | 5.7 (1.06) |

*How sure the physician is about his or her choice on a scale of 1 to 7, where 1 means 'not sure at all' and 7 means 'completely sure'.

*IV* intravenous, *SC* subcutaneous, *SD* standard deviation
